# Supplementary material for: WordCommentsAnalyzer: A windows software tool for qualitative research
Source: F1000Res. 2018 Sep 4;7:536. Originally published 2018 May 3. [Version 2] doi: 10.12688/f1000research.14819.2 (PMC6137411; doi:10.12688/f1000research.14819.2)
Supplement: Supplementary file 3 [file f1000research-7-17614-s0002.tgz › b2a79496-4ada-47c4-9682-a99ce867836d.zip!/Eight Iranian Health Journals/Salmand_Iranian Journal of Ageing _34.docx]

© Salmand_Iranian Journal of Ageing . This work is licensed under CC BY-NC 4.0 (https://creativecommons.org/licenses/by-nc/4.0/).

Volume & Issue: دوره 5 - شماره 1

Title: تجارب سالمندان تهرانی در زمینه اوقات فراغت و تفریحات

http://salmandj.uswr.ac.ir/article-1-295-fa.html

اهداف: این مطالعه با هدف تبیین مضامین الگوهای مختلف تفریح و گذران اوقات فراغت سالمندان تهرانی طراحی و اجرا گردید. مواد و روش‌ها: برای دستیابی به این هدف، با 17 سالمند در دامنه سنی 86-65 سال در سال 1388 در تهران مصاحبه نیمه ساختار یافته انجام شد. تجزیه و تحلیل داده ها با استفاده از روش تحلیل محتوی کیفی (کانونشنال) صورت پذیرفت. یافته‌ها: در این پژوهش، پنج مضمون اصلی از دیدگاه مشارکت کنندگان به دست آمد. این مضمون ها عبارت بودند از: 1. فعالیت های رایج اوقات فراغت در سالمندی؛ 2. موانع استفاده از اوقات فراغت؛ 3.  نقش خانواده در اوقات فراغت سالمند؛ 4. تاثیر گردش بر زندگی سالمند؛ 5. راهکارهای بهبود نحوه گذران اوقات فراغت سالمند. برای هر یک از این مضامین چند زیر مضمون مشخص شد. نتیجه‌گیری: یافته های این مطالعه، با توجه به تجربیات مشارکت کنندگان از مفهوم مورد مطالعه، نشان داد که سالمندان از الگوی اوقات فراغت رضایت بخش برخوردار نیستند.

Title: اثر توانبخشی شناختی در کاهش نقایص شناختی سالمندان مبتلا به دمانس آلزایمر

http://salmandj.uswr.ac.ir/article-1-296-fa.html

اهداف: هدف پژوهش حاضر، بررسی تاثیر توان بخشی شناختی در کاهش نقایص شناختی سالمندان مبتلا به دمانس آلزایمر بود. مواد و روش‌ها: این پژوهش با یک طرح شبه تجربی، با روش طرح های تک آزمودنی و A-B با گروه کنترل و دسته درون سری ها انجام شد. 8 بیمار مبتلا به آلزایمر خفیف و خفیف تا متوسط، با روش نمونه گیری در دسترس انتخاب و به دو گروه آزمایش و گواه تقسیم شدند. مداخله به مدت 12 جلسه اجرا شد (هفته ای 2 جلسه، به مدت 45 دقیقه تا یک ساعت). به منظور بررسی تاثیر مداخلات (توان بخشی حافظه و توجه)، کلیه آزمودنی ها جلسه اول و آخر توسط پرسشنامه معاینه مختصر وضعیت روانی (MMSE) و مقیاس حافظه وکسلر (WMS-O) مورد ارزیابی قرار گرفتند. تجزیه و تحلیل داده های پژوهش، هم در سطح بین فردی (زوج های همتا) و هم به صورت گروهی (با استفاده از محاسبه درصد بهبودی و اندازه اثر) صورت گرفت. یافته‌ها: تحلیل های آماری نشان داد که آزمودنی های گروه آزمایش، چه در مقایسه با آزمودنی همتا و چه در مقایسه گروهی، نسبت به خط پایه، در نمرات تغییر و بهبودی نشان دادند. اندازه اثر محاسبه شده برای کارکرد شناختی و کارکرد حافظه گروه آزمایش به ترتیب (d=1/22) و (d=2/54) بود، که نشان دهنده تغییر در گروه آزمایش نسبت به گروه گواه بود. نتیجه‌گیری: توانبخشی حافظه و توجه در کاهش نقایص شناختی سالمندان مبتلا به مانس نوع آلزایمر تاثیر دارد.

Title: مقایسه تغییرات وابسته به سن بر عملکرد تعادلی در جوانان و سالمندان

http://salmandj.uswr.ac.ir/article-1-292-fa.html

اهداف : هدف از مطالعه حاضر، بررسی تاثیرات سالمندی بر عملکرد تعادلی (ایستادن روی یک پا) تحت شرایط حسی مختلف می باشد. مواد و روش‌ها: مطالعه مقطعی – مقایسه ای حاضر، در 20 فرد جوان با دامنه سنی (22/75±2/29 سال)، و 20 فرد سالمند با دامنه سنی (65/1±4/16 سال)، انجام شد. مدت زمان ایستادن افراد در تکلیف تعادلی (ایستادن روی یک پا) در سه وضعیت حسی در حضور و در غیاب بینایی روی سطح سخت و در غیاب بینایی روی سطح فوم توسط کرنومتر ثبت گردید. آزمون تی مستقل، جهت مقایسه هر یک از وضعیت های حسی در دو گروه، آزمون تحلیل واریانس یک طرفه برای بررسی هر سه وضعیت حسی در هر گروه و آزون تحلیل واریانس دو طرفه جهت تعیین کارایی تکلیف تعادلی در دو گروه، مورد استفاده قرار گرفت. یافته‌ها: تفاوت معناداری میان جوانان و سالمندان در تکلیف تعادلی تحت هر یک از سه وضعیت حسی مشاهده شد (P<0/05). نتیجه‌گیری: نتایج بیانگر اهمیت درون دادهای حسی دخیل در کنترل وضعیتی با افزایش سن است. به علاوه، می توان از تست ایستادن بر روی یک پا، به عنوان یک ابزار بالینی در محیط های کلینیکی و نیز در مطالعات برای بررسی تاثیرات سالمندی استفاده نمود.

Title: طراحی و روانسنجی پرسش‌نامه سوءرفتار نسبت به سالمندان در خانواده

http://salmandj.uswr.ac.ir/article-1-290-fa.html

اهداف: پژوهش حاضر با هدف طراحی و تعیین ویژگی های روان سنجی پرسشنامه ای متناسب با معیارهای فرهنگی جامعه ایرانی جهت بررسی سوء رفتار با سالمندان در خانواده انجام شده است. مواد و روش‌ها: مطالعه حاضر یک پژوهش روش شناختی است که در 3 مرحله انجام گرفت. در مرحله اول محققین با استفاده از روش پدیدارشناسی به تبیین مفهوم سوء رفتار با سالمندان در خانواده پرداختند. در مرحله دوم، عبارات پرسشنامه، بر اساس مصاحبه های بدون ساختار با قربانیان سالمندآزاری و استقراء از گروه متخصصین، تدوین گردید. سپس در مرحله سوم، ویژگی های روانسنجی پرسشنامه با بهره گیری از روانی صوری، روایی محتوا، روایی سازه، همسانی درونی و ثبات، مورد بررسی قرار گرفت. در مرحله اخیر، 325 سالمند تهرانی به صورت تصادفی از بیمارستان ها و کانون های جهاندیدگان منتخب شهر تهران، انتخاب شده، در مطالعه شرکت کردند. یافته‌ها: بر اساس نتایج حاصل از مرحله اول و دوم مطالعه، پرسشنامه اولیه شامل 78 عبارت ارایه شد. پس از تعیین روایی صوری و محتوا به صورت کمی و کیفی، عبارات پرسشنامه به 49 عبارت کاهش یافت. نتیجه تحلیل عاملی اکتشافی در این پرسشنامه، بیانگر وجود 8 عامل بود: غفلت مراقبتی، سوء رفتار روان شناختی، سوء رفتار جسمی، سوء رفتار مالی، سلب اختیار، طردشدگی، غفلت مالی و غفلت عاطفی. این 8 عامل در مجموع 79/93 درصد از واریانس کل آزمون را تبیین می نمودند. نتایج روش تحلیل مقایسه ای گروه های شناخته شده در پارامترهای وضعیت عملکردی و بستری مجدد، نمایانگر تفاوت های قابل ملاحظه ای بین گروه ها بود. محاسبه ضریب آلفای کرونباخ، موید همسانی درونی بالای پرسشنامه (0/9-0/975) و ثبات از طریق آزمون مجدد 0/99 بود. نتیجه‌گیری: یافته ها، حاکی از آن است که پرسشنامه حاضر با عنوان «پرسشنامه بررسی سوء رفتار با سالمند در خانواده» با 49 عبارت، و وجود ویژگی هایی نظیر طراحی ابزار، بر اساس درک مفهوم سوء رفتار از دیدگاه سالمند ایرانی و مبتنی بر تجربیات زیسته آنان، تبیین کلیه اشکال سوء رفتار با سالمند در خانواده، نمره گذاری ساده، پایایی و روایی مناسب و قابلیت به کارگیری در موقعیت های مختلف، از سوی ارایه دهندگان خدمات بهداشتی، از جمله پرستاران، ابزاری مناسب جهت بررسی سوء رفتار با سالمند در خانواده های ایرانی است.

Title: نیازهای بیماران سالمند بستری در بیمارستان

http://salmandj.uswr.ac.ir/article-1-293-fa.html

اهداف: این مطالعه به منظور تبیین نیازهای سالمندان بستری در بیمارستان انجام شد. مواد و روش‌ها: پژوهش حاضر با رویکرد کیفی و با کاربرد روش تحلیل محتوا انجام شده است، بیست و هفت بیمار سالمند 65 تا 81 ساله بستری در سه بیمارستان آموزشی شهر تهران با روش نمونه گیری هدفمند مورد مطالعه قرار گرفتند. داده ها با استفاده از مصاحبه های بدون ساختار و عمیق تا رسیدن به سطح اشباع اطلاعات، ادامه یافت و سپس بر اساس روش مرحله ای کاول، تحلیل گردید. یافته‌ها: داده ها در 6 طبقه قرار گرفت که نیازهای تجربه شده بیماران سالمند را به تصویر می کشند. این طبقات عبارتند از: برخورداری از امکانات رفاهی اولیه، امکان مشارکت در مراقبت، درک مبتنی بر همدلی، تکریم و حفظ شان و منزلت، تامین اطلاعات ضروری، القای امید و خوش بینی. نتیجه‌گیری: یافته ها، بیانگر ادراکات منفی و مثبت و نیز مشکلات متعدد تجربه شده بیماران سالمند بود. این یافته ها می توانند به درک بهتر مشکلات مرتبط با تجربه بستری شده سالمند در بیمارستان، شناسایی مداخلات موثر در کاهش عوامل استرس زا و تعدیل تاثیر این تجربه بر سالمند، کمک کنند. این تحقیق، ضرورت آموزش تیم مراقبت به خصوص پرستاران را بر اساس نیازهای بیماران سالمند مورد تاکید قرار می دهد.

Title: رابطه عمل به باورهای دینی و رضایت از زندگی در بین سالمندان

http://salmandj.uswr.ac.ir/article-1-297-fa.html

اهداف: در پژوهش حاضر رابطه بین عمل به باورهای دینی و رضایت از زندگی در بین سالمندان شهر سبزوار مورد بررسی قرار گرفت. مواد و روش‌ها: پژوهش حاضر، در چارچوبی مطالعات پیمایشی انجام گرفت تعداد 301 نفر از سالمندان شهر سبزوار به عنوان نمونه مورد بررسی به روش خوشه ای انتخاب شدند. ابزار گردآوری داده ها عبارت بودند از: پرسشنامه میزان عمل به باورهای دینی (معبد) و مقیاس رضایت از زندگی (SWLS) برای تحلیل داده ها از آزمون های آماری نظیر ضریب همبستگی، آزمون تی و رگرسیون چند متغیره استفاده شد. یافته‌ها: چهار حوزه اصلی مقیاس معبد، یعنی عمل به واجبات، عمل به مستحبات، عضویت در گروه های مذهبی و مداخله دادن مذهب در تصمیم گیری و انتخاب، رابطه معنی دار با رضایت از زندگی داشتند. تفاوت بین نمره رضایت از زندگی و عمل به باورهای دینی زنان و مردان سالمند معنی دار بود. همچنین بین رضایت از زندگی سالمندان و تحصیلات آنها رابطه معنی داری وجود داشت. نتیجه‌گیری: رضایت از زندگی در بین سالمندانی که عمل به باورهای دینی دارند، بیشتر است و این با آموزه های دین مبین اسلام همسوست.

Title: بررسی ویژگی‌های مرتبط با سلامت سالمندان جمعیت منطقه مینودر شهر قزوین به منظور طراحی مداخلات پژوهشی در سال 1387

http://salmandj.uswr.ac.ir/article-1-294-fa.html

اهداف: این مطالعه با هدف تعیین علل مراجعه سالمندان برای دریافت خدمات درمانی و علل بستری و شناسایی رضایت آنان از بیمه درمانی و ارزیابی اولویت های پژوهشی مرتبط با سلامت آنان انجام شد. مواد و روش‌ها: در این مطالعه توصیفی –تحلیلی، اطلاعات طی یک هفته جمع آوری شد و 119 نفر را با روش نمونه گیری خوشه ای از افراد بالاتر از 60 سال در محلات منطقه مینودر توسط رابطین بهداشتی محلات و به صورت خانه به خانه و تکمیل پرسشنامه انتخاب کردند. پرسشنامه شامل 25 سوال بود و برای آنالیز داده ها از آمار توصیفی و استنباطی در محیط نرم افزار SPSS/15 استفاده شد. یافته‌ها: شایع ترین علل مراجعه به پزشک در بین سالمندان، بیماری های استخوان و مفاصل (22/6%) بیماری های قلب و عروق (15/5%) بیماری های گوارشی و غدد (15/5%) و میانگین دفعات استفاده از پروتز برای هر یک نفر 0/98 بار بود. میانگین دفعات مراجعه سالمندان برای یک سال به پزشک و داروخانه (10/7 بار)، دفعات بستری در بیمارستان (0/4 بار)، طول مدت اقامت در بیمارستان (3/9 روز)، هزینه خری دارو و وسایل پزشکی، (برای هر یک بار 60330 ریال) بود. شایع ترین علل بستری سالمندان در بیمارستان به ترتیب بیماری های قلبی عروقی (20/3%)، جراحی ها (20/1%)، عفونی و داخلی (13/3%) و چشم (8/8%) بود. این مطالعه، نشان داد که %64/2 از افراد بیمه شده، پزشک بیمه و %76/4 آنان درمانگاه دولتی را برای دریافت خدمات درمانی خود انتخاب کرده بودند و مهم ترین علت این انتخاب در هر دو مورد، هزینه بود. 44/5% از سالمندان تحت مطالعه، در مقابل افزایش کیفیت خدمات درمانی دریافتی، با افزایش پرداخت سرانه بیمه موافق و %55/5 با این افزایش مخالف بودند. نتیجه‌گیری: الگوی شایع ترین علل مراجعه به پزشک و علل بستری و الگوی ارایه خدمات درمانی از سال های قبل تا زمان اجرای این مطالعه در سالمندان تغییر محسوسی نداشته، در عین حال میانگین دفعات مراجعه به پزشک و داروخانه و بستری در سالمندان، به نحو محسوسی بیشتر از غیر سالمندان بود و هزینه درمان نیز افزایش یافته بود، بنابراین موضوعاتی برای طراحی مداخلات پژوهشی برای ارتقای سطح سلامت سالمندان در منطقه مینودر شهر قزوین پیشنهاد می شود.

Title: ارائه انگاره های محیطی مؤثر بر شکل‌گیری فضاهای عمومی مشوق سالمندی موفق با تأکید بر ترجیحات سالمندان شهر شیراز

http://salmandj.uswr.ac.ir/article-1-291-fa.html

اهداف: هدف این پژوهش، ارایه انگاره های محیطی است که بر میزان حضور و مشارکت سالمندان در فضاهای عمومی شهری تاثیر داشته، متضمن دستیابی به الگوی سالمندی موفق در فضاهای عمومی شهری است. مواد و روش‌ها: این پژوهش، از نوع توصیفی‌تحلیلی و در بستری پیمایشی بود. جامعه آماری این پژوهش، سالمندان بالای 65 سال شهر شیراز بودند که در فضاهای عمومی حضور و مشارکت داشتند و در این فضاها به فعالیت های مختلف می پرداختند. داده های این پژوهش از دو گروه نمونه 50 نفری با مصاحبه عمیق (مطالعه مقدماتی) و 144 نفری با پرسشنامه محقق ساخته، جمع آوری شد. نمونه گیری به روش تصادفی ساده انجام شد. همچنین از نرم افزارهای SPSS و LISREL به عنوان ابزار تحلیل و ارزیابی داده ها و از مدل ساختاری تحلیل عاملی تاییدی و روش تحلیل حداقل مربعات غیر وزنی به عنوان روش تحلیل داده ها بهره گرفته شد. یافته‌ها: انگاره های زیر در ارزش گذاری فضاهای عمومی شهری از دید سالمندان برای حضور و مشارکت تاثیر گذارند: 1. راه یابی و دسترسی (دسترسی عمومی به فضا T=4/81)؛ 2. ایمنی و امنیت (وقوع کم جرم و جنایت T=14/09)؛ 3. آسایش (مبلمان مناسب و طراحی شده T=5/59)؛ 4. پویایی و جذابیت (وجود کاربری متنوع T=3/94)؛ 5. هم آوایی با طبیعت (پاکیزگی و عدم وجود آلودگی T=5/44)؛ 6. رمز گرایی و ابهام (وجود نقاط قابل کشف T=7/24)؛ 7. هویت (رنگ و مصالح هماهنگ با محیط قرارگیری T=3/37)؛ 8. آزادی (آزادی انجام دادن فعالیت های متنوع T=9/49)؛ 9. رویدادپذیری (بروز رویدادهای فرهنگی T=4/65)؛ 10. بوم گرایی (هماهنگی با محیط پیرامونی T=4/44)؛ 11. وحدت (پراکندگی کم عناصر فضا T=26/72)؛ 12. نظم (نظم در کلیات و جزییات فضا (T=15/79 و 13. خاطره ذهنی (طراحی فضای عمومی مطابق با آداب و رسوم محیط قرارگیری T=7/76). نتیجه‌گیری: به نظر می رسد شناخت و درک نیازهای مختلف سالمندان و تلاش در جهت پاسخگویی به این نیازها و احترام به ترجیحات آنها در طراحی و بهسازی فضاهای عمومی شهری، می تواند در زندگی خوب سالمندی و بهبود کیفیت و امید به زندگی آنها و دستیابی به اهداف سالمندی موفق، موثر باشد.
